# KORRIGAN1 Interacts Specifically with Integral Components of the Cellulose Synthase Machinery

**DOI:** 10.1371/journal.pone.0112387

**Published:** 2014-11-10

**Authors:** Nasim Mansoori, Jaap Timmers, Thierry Desprez, Claire L. A. Kamei, Dianka C. T. Dees, Jean-Paul Vincken, Richard G. F. Visser, Herman Höfte, Samantha Vernhettes, Luisa M. Trindade

**Affiliations:** 1 Plant Breeding, Wageningen University and Research Centre, Wageningen, The Netherlands; 2 Graduate School Experimental Plant Sciences, Wageningen University, The Netherlands; 3 Carbohydrate Research Centre, Wageningen University and Research Centre, Wageningen, The Netherlands; 4 Laboratoire de Biologie Cellulaire, Institute Jean-Pierre Bourgin, Institut National de la Recherche Agronomique, Versailles, France; UMass, United States of America

## Abstract

Cellulose is synthesized by the so called rosette protein complex and the catalytic subunits of this complex are the cellulose synthases (CESAs). It is thought that the rosette complexes in the primary and secondary cell walls each contains at least three different non-redundant cellulose synthases. In addition to the CESA proteins, cellulose biosynthesis almost certainly requires the action of other proteins, although few have been identified and little is known about the biochemical role of those that have been identified. One of these proteins is KORRIGAN (KOR1). Mutant analysis of this protein in *Arabidopsis thaliana* showed altered cellulose content in both the primary and secondary cell wall. KOR1 is thought to be required for cellulose synthesis acting as a cellulase at the plasma membrane–cell wall interface. KOR1 has recently been shown to interact with the primary cellulose synthase rosette complex however direct interaction with that of the secondary cell wall has never been demonstrated. Using various methods, both *in vitro* and *in planta*, it was shown that KOR1 interacts specifically with only two of the secondary CESA proteins. The KOR1 protein domain(s) involved in the interaction with the CESA proteins were also identified by analyzing the interaction of truncated forms of KOR1 with CESA proteins. The KOR1 transmembrane domain has shown to be required for the interaction between KOR1 and the different CESAs, as well as for higher oligomer formation of KOR1.

## Introduction

Cellulose is synthesized by a large rosette terminal complex, which comprises at least three different cellulose synthases (CESAs). On the basis of *Arabidopsis thaliana* mutational analysis, CESAs are not assumed to work alone. Some proteins were predicted to be associated with the cellulose synthase complex, although their direct interaction has never been demonstrated. One of these proteins is KORRIGAN (KOR1), a membrane-bound endo-1,4-β-D-glucanase with a single transmembrane domain and two putative polarized targeting signals in the cytosolic tail [1], [2]. KOR1 is thought to be required for cellulose synthesis acting at the plasma membrane–cell wall interface in plants and other organisms such as bacteria [3], [4], however the precise role of KORRIGAN in cellulose biosynthesis is still unknown. KORRIGAN is suggested to have a proof-reading activity and relieve stress generated during the assembly of glucan chains in cellulose microfibrils by means of hydrolyzing disordered amorphous cellulose [5]. The enzyme heterologously produced in *Pichia pastoris* cleaves non substituted but non-crystalline 1,4-β–linked glucan chains and shows no activity against xyloglucans [3], [6], [7]. A member of the endo-1,4-β-glucanase family has also been proposed to mediate the transfer of a glucose residue to a growing β-glucan chain during cellulose synthesis in *Agrobacterium tumefaciens* and *Acetobacter xylinum* [4], [8].

Besides *KOR1*, two additional genes encoding membrane-anchored endoglucanases (EGase) have also been characterized in *A. thaliana* [3]. The *KOR1* gene is the most widely expressed membrane-anchored EGase throughout various plant tissue while *KOR2* and *KOR3* are active in specific cell types. Expression of a GUS reporter gene driven by the endogenous promoters of *KOR2* and *KOR3* have shown that *KOR2* is active in trichomes and floral organs while *KOR3* is active in developing root hairs. Microsomal fractioning demonstrated that KOR1 is present in the tonoplast, the Golgi apparatus [9], the cell plate [2] and importantly in the plasma membrane [1] where cellulose biosynthesis occurs [10], [31].

The dwarf mutant *korrigan* (*kor1-1)* showed defects in some aspect of cell wall loosening in primary cell wall biosynthesis [1]. Other *KOR* mutations (*kor1–2*) have shown to cause the formation of aberrant cell plates and incomplete cell walls [2]. Morphological and chemical analysis of KOR temperature sensitive, single base pair mutants (*acw1* altered cell wall and *rsw2* root swelling) showed abnormal plant morphology, defects in primary cell wall formation, reduced cellulose content, increased pectin synthesis, and aberrant cell division similar to that found in the *CESA1* mutant *rsw1* [1], [2], [11], [12], [13]. Cellulose synthesis, which occurs concomitantly with cell wall loosening during cell elongation is impaired in the temperature sensitive elongation deficient *acw1* mutant grown at 31°C and shows a 40% reduction in crystalline cellulose content compared to the wild type [12], [15]. Significant reduction in cellulose production was seen in seedlings of double mutants of cellulose synthase (*rsw1*) and *KOR* (*rsw2*) in comparison to either of the single mutants which demonstrates that cellulose biosynthesis in the primary cell walls of plants requires both a glycosyl transferase and glycosyl hydrolase providing further evidence that KOR has a key role in cellulose deposition in the primary cell wall [13]. Recently, direct interaction between KOR1 and primary cell wall CESA proteins has been reported supporting a new model in which KOR1 is an integral part of the CSC, where it plays a role in the synthesis of cellulose and also in the intracellular trafficking of the CSC in the primary cell wall [14].

Reports have indicated that KOR also plays a role in secondary cell wall development. An accumulation of a *KOR* homolog was observed during secondary cell wall deposition in cotton [17]. Two independent mutations (*irx2-1* and *irx2-2*) in the *KOR1* genes showed similar phenotypes as the *irx* mutants, i.e. collapsed xylem cell walls due to reduced cellulose synthesis in the secondary cell wall in the base of mature stems [16]. It must also be mentioned that the *kor1-1* mutants that were reported to be primary cell wall mutants also had severely collapsed xylem cells similar to the *irx2* mutants [16]. Targeted down-regulation of the *KOR* genes from hybrid poplar led to moderate to severe defects in plant growth, an irregular xylem (*irx*) phenotype commonly associated with KOR and other secondary cellulose-specific mutants in *A. thaliana* [17].

Since KOR appears to be associated with cellulose synthesis and direct association has been detected with the primary cellulose synthase protein complex [14], it is important to determine whether or not there is also direct interaction with the CESA proteins in the secondary cell wall. In this study, using the membrane-based yeast two hybrid system (MbYTH; [18], [19]) and the bimolecular fluorescence complementation method (BiFC), it is shown that KOR1 interacts specifically with CESAs involved in the deposition of secondary cell walls. The data demonstrate that the interaction between KOR1 and the CESA proteins is specific and probably occurs at the plasma membrane.

## Materials and Methods

### Constructs for the MbYTH system

**Figure 1:**
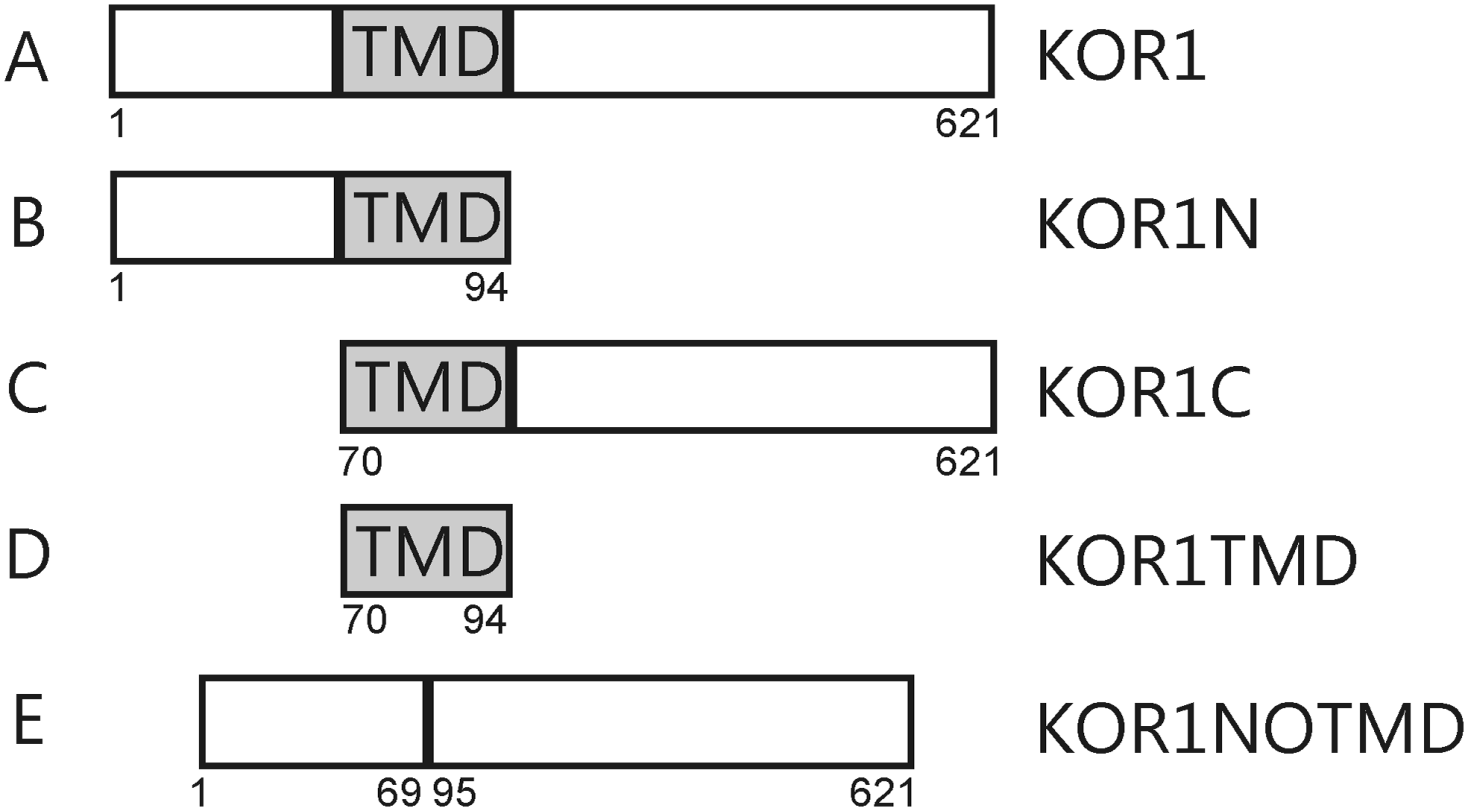
Representation of different truncated KORRIGAN proteins used in this study. A) KOR1 is the complete protein (AA 1 to 621), B) KOR1N: the N-terminal part with the transmembrane domain (AA 1 to 94), C) KOR1C: transmembrane domain (TMD) plus the C-terminal part of KOR1 (AA 70 to 621), D) KOR1TMD only the TMD of KOR1 (AA 70 to 94), E) KOR1NOTMD is the KOR1 protein without the TMD, in which the N-terminus is fused directly to the C-terminal part (AA 1 to 69+95 to 621). Bellow each fragment the aminoacid numbers are indicated.

The constructs for the MbYTH system concerning the primary and secondary CESAs were generated as described previously [20], [19]. The full-length AtKOR1 (RAFL05-02-G06) cDNA was obtained from the Riken Bioresource center [21], [22]. Several truncated forms of KOR1, KORRIGAN transmembrane domain (KORTMD; AA 70 to 94, Figure 1D), the TMD with the C-terminal portion of the protein (KORC; AA 70 to 621, Figure 1C), the N-terminal part of the protein together with the TMD (KORN; Amino Acids 1 to 94, Figure 1B) and only the cytosolic part (N-terminal and C-terminal) of the protein (KORNOTMD AA 1 to 69+95 to 621, Figure 1E) were also made (Figure 1). The complementary DNA (cDNA) of the mentioned genes were amplified by PCR using the Phusion DNA Polymerase (Finnzymes, Helsinki, Finland) with the primers (Table S1). The resulting PCR-products were digested and ligated into the pTFB1 vector (Bait) and the pADSL-Nx vector (Prey; Dualsystems Biotech AG). Bait and prey expression is regulated by the TEF1 and ADH1 promoter, respectively. The sequences of the inserts were confirmed by Sanger sequence analysis. Both the bait and prey protein were fused N-terminally to the Cub-TF reporter cassette of the vector pTFB1 and NubG cassette of the vector pADSL-Nx respectively.

### Membrane based yeast two hybrid screen

The interactions between the CESAs and KORRIGAN1 were assayed with the split-ubiquitin membrane-based yeast two-hybrid [23], [24] using yeast NYM51 strain of *Saccharomyces cerevisiae* in the Split Ubiquitin System kit (Dualsystems Biotech AG). Interactions were performed according to supplier instructions (DUAL membrane Kit 1) and were tested with KOR1 fused to the C-terminal part of the ubiquitin (Cub) and the transcription factor (bait), whereas the CESA1, 3 and 6 proteins were fused to the N-terminal part of the ubiquitin (Nub; preys). The bait and prey constructs were co-transformed into the yeast strain NMY51 (Dualsystems Biotech AG) according to the provided transformation procedure (DUAL membrane Kit 1). Upon interaction between the bait and the prey the transcription factor (TF) is released into the nucleus where it activates reporter genes allowing the yeast to grow on selective medium lacking leucine and tryptophan (SD med. -L-T), and subsequently grown at 30°C for three days. To quantify the interactions between different preys 100 colonies of each combination were spotted onto selection medium containing the appropriate amount of 3-ammonium-triazole (3-AT) and grown at 30°C for three days. The number of spots grown was then counted.

### Constructs for Split-YFP

The full-length cDNA of the primary and secondary *CESA* genes, *KORRIGAN1* as well as the truncated forms of KOR1 were generated through Phusion DNA Polymerase (Finnzymes, Helsinki, Finland) with suitable primers ([24], [17]; Table S1). Coding sequences of the genes were cloned into the Gateway-compatible destination vectors pBIFc-2 and pBIFc-3 plasmids regulated by the constitutive 35S promoter [27]. The N-terminal and the C-Terminal fragments of YFP were both fused to the N terminus of the coding sequences of the CESAs. As a positive control, the aquaporin PIP2-1 [24], [25], [26] was used, as Aquaporins are known to form homotetramers in the plasma membrane [28], [29]. As negative controls, the N-terminal fragment of YFP (Y/N) was co-expressed with the C-terminal of YFP (Y/C) to check for autofluorescence as well as with the KOR1 construct (YC-KOR1).

### Statistical Analysis

Statistical significance of differences in interactions were evaluated by using the unpaired, two-tailed *t-*test. Probability levels p≤0.05 were taken as statistically significant.

### Bimolecular Fluorescence Complementation screen

Using this system, the interaction between the primary CESAs and KOR1 were tested in leaves of 3-week-old *Nicotiana benthamiana* plants. Two YFP fragments, either Y/N or Y/C, each linked to the N-terminus of the proteins, were transiently expressed in the plant by infiltration as described [30]. Upon interaction between the two proteins, the fragments restore fluorescence, which can be detected. YFP fluorescence was detected 3 days after infiltration by using the 514-nm laser line of a SP2 AOBS confocal laser scanning microscope (Leica, Solms, Germany) equipped with an argon laser. To check the YFP reconstitution, spectral analysis was performed with the 496-nm laser line. The fluorescence with all constructs was detected at the same photo-multiplier tube (PMT) gain settings (760), except for the negative interactions for which the PMT was increased up to 880.

## Results

### KORRIGAN1 interacts specifically with CESAs for both primary and secondary cell wall

**Figure 2:**
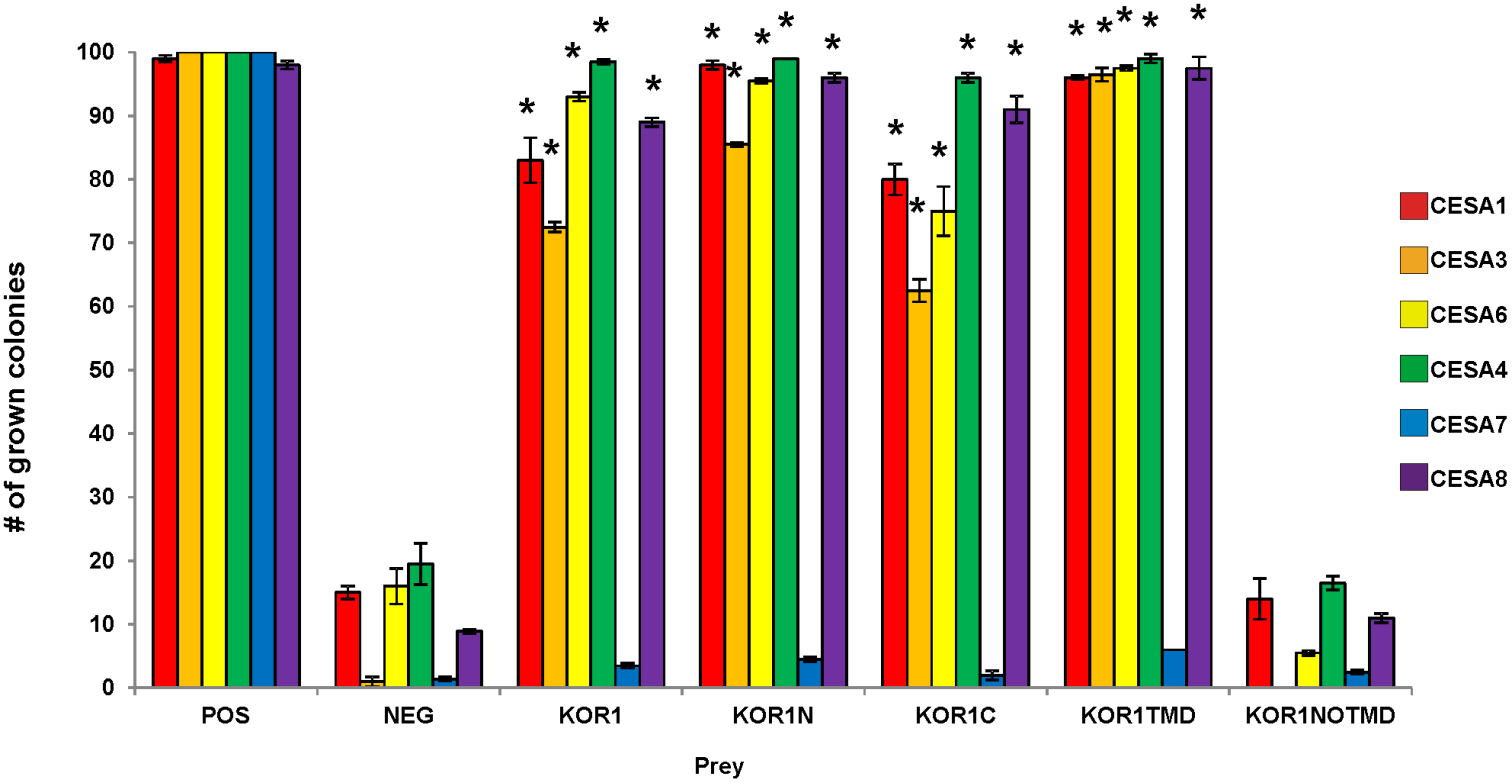
Interactions between the different KOR1 domains and the different CESA proteins using the Membrane-based Yeast Two Hybrid. The bars represent the percentage of yeast colonies grown for 3 days on selective medium at 30°C. The different CESA proteins were expressed in yeast as baits and the different KOR1 protein domains as preys. ALG5 protein fused to wild-type ubiquitin domain was used as a positive control (POS) and NubG, fused to mutated ubiquitin domain as neg. control (NEG). The results are representative of three independent experiments, n = 3, Asterisks indicate statistically significant differences compared with the negative control (Student’s *t* test, *P<0.05).

The interactions between KOR1 and three members of the primary cell wall cellulose-synthesizing rosette complex (CESA1, CESA3 and CESA6) have been demonstrated *in vitro* using the membrane based yeast two hybrid system [14]. The results indicated that KOR1 is able to interact with all three of the primary CESA proteins as yeast colonies were able to grow on selective medium with KOR1 as bait and the primary CESAs as prey. Here the converse experiments, in which the different CESA proteins were the bait and the KOR1 the prey were carried out to confirm the findings, and showed that all combinations were able to induce the reporter genes, allowing the yeast to grow on selective medium (Figure 2). The lack of growth in the negative control indicated that the interaction with KOR1 was specific as an unrelated protein expressed as prey is not able to activate the system. We conclude that KOR1 can interact with CESA1, CESA3, and CESA6 *in vitro*.

**Figure 3:**
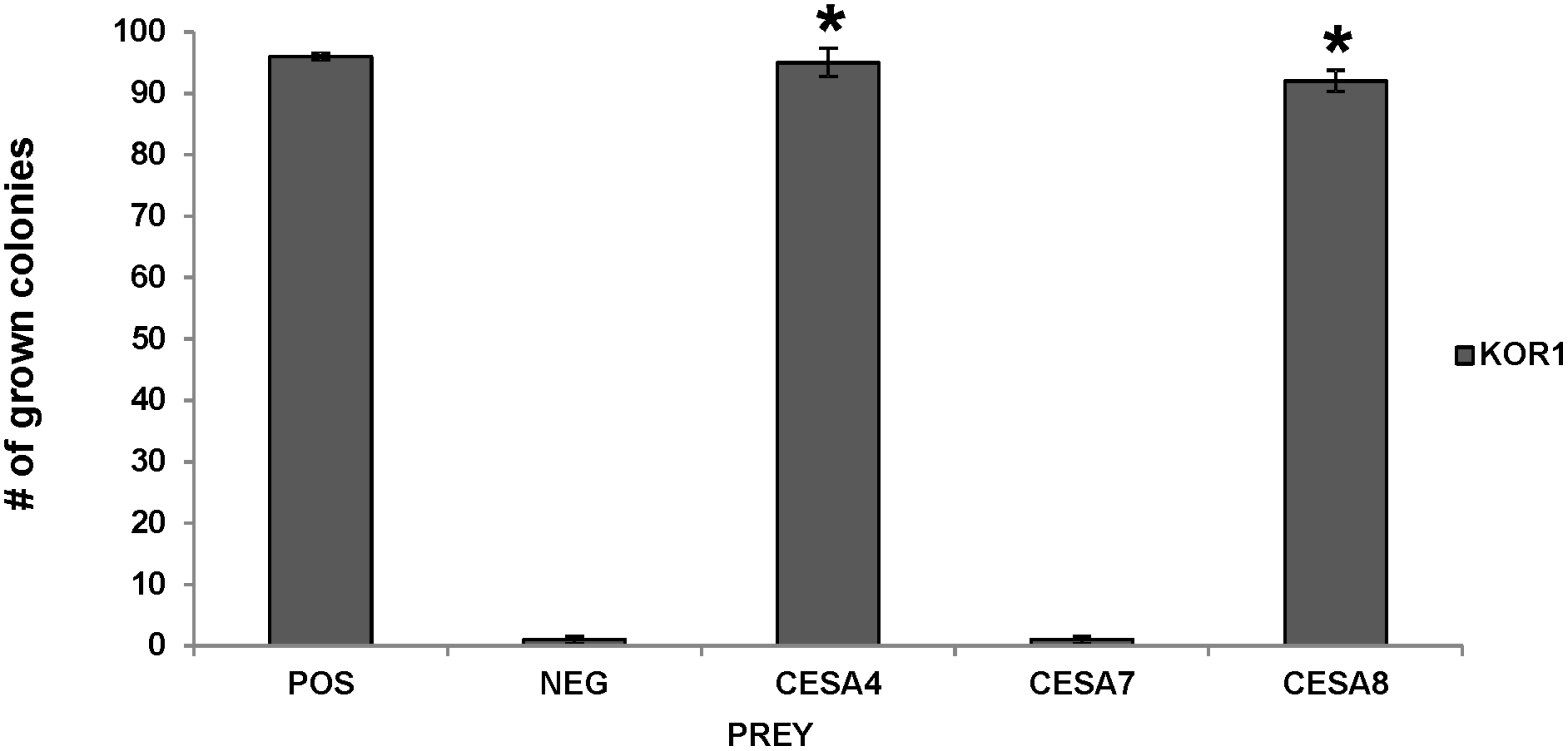
Interactions between KOR 1 and the different secondary CESA proteins using the Membrane-based Yeast Two Hybrid. The bars represent the percentage of yeast colonies grown for 3 days on selective medium at 30°C. KOR1 was expressed in yeast as bait and the different secondary CESA proteins as prey (as indicated in the legend). ALG5 protein fused to wild-type ubiquitin domain was used as a positive control (POS) and NubG, fused to mutated ubiquitin domain as neg control (NEG). The results are representative of three independent experiments, n = 3, Asterisks indicate statistically significant differences compared with the negative control (Student’s *t* test, *P<0.05).

Reduction in cellulose content of the secondary cell wall in the KOR1 (*irx*) mutants also link KOR1 to cellulose synthesis in the secondary cell wall. To test a possible interaction between KORRIGAN and the secondary CESA proteins (CESA4, CESA7 and CESA8) the KOR1 was expressed as bait in combination with the secondary CESA as prey. As a negative control, we tested KOR1 as bait with an unrelated protein AGL5 as prey (Timmers et al., 2009). The yeast strains failed to grow in multiple repetitions of this experiment, demonstrating that a specific interaction between the bait and prey is required to activate the system (Figure 3). The combination of KOR1 with CESA4 or CESA8 activated the reporter genes, and therefore was able to grow on selective medium whereas no growth was detected when KOR1 and CESA7 were tested (Figure 3). The results were further confirmed by the interaction between the CESA proteins as bait and the KOR1 protein as prey (Figure 2).

**Figure 4:**
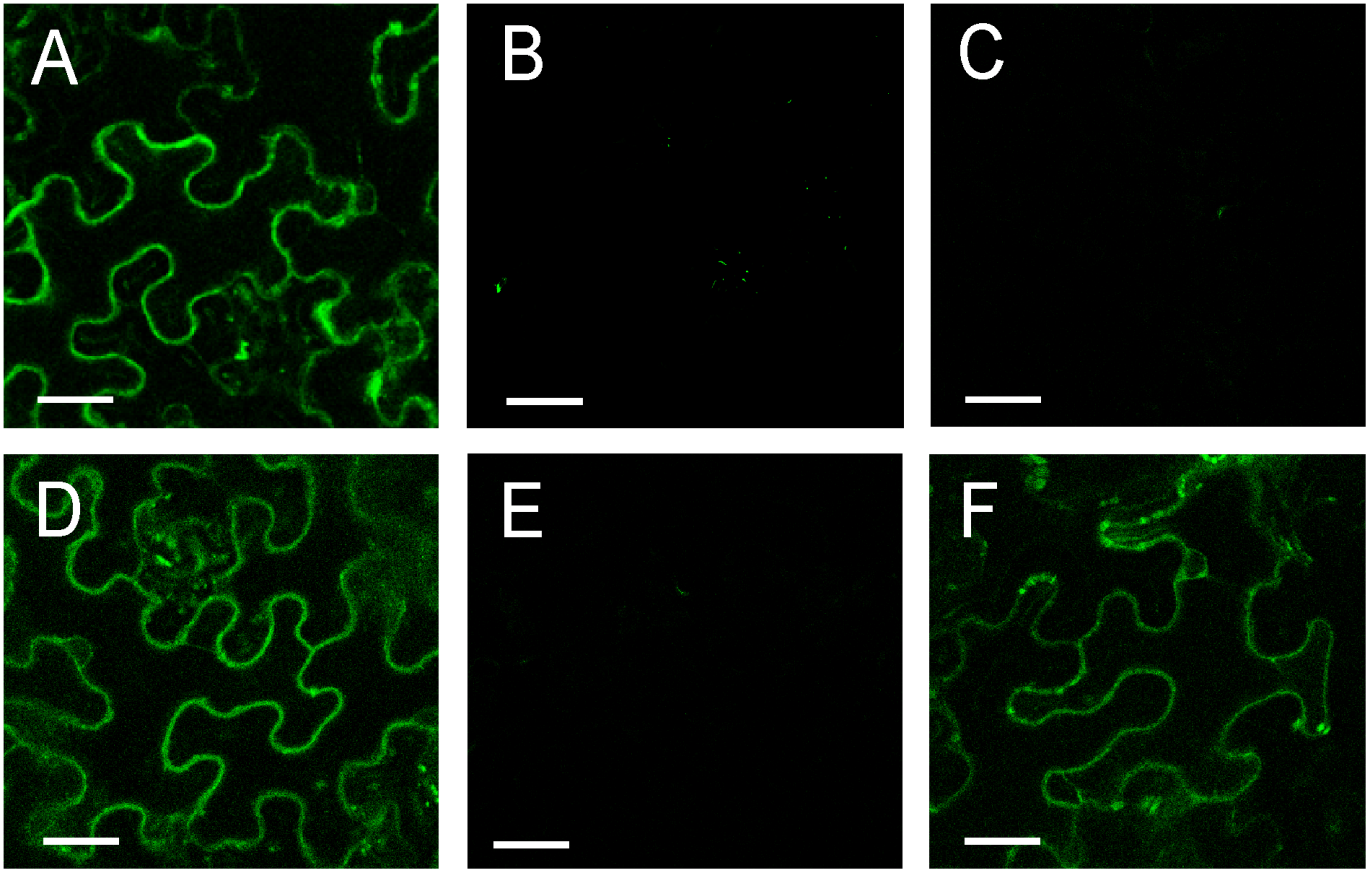
Bimolecular fluorescence (BiFC) analysis of dimerization between KORRIGAN and various CESA proteins. The proteins were transiently expressed in *N. benthamiana* leaf epidermal cells. (A) positive control YN-PIP2/YC-PIP2, negative controls (B) YN/YC-KOR1 and (C) YN/YC, (D)YN-KOR1/YC-CESA4, (E) YN-KOR1/YC-CESA7, (F) YN-KOR1/YC-CESA8. The results are representative of three independent experiments, n = 3. The scale bar is 100 µm.

The physical interaction of KOR1 with the secondary CESA proteins was also tested *in planta*. Both KOR1 and the different secondary CESA proteins were fused to fragments of the YFP and expressed in *Nicotiana benthamiana* leaves in different combinations. Restored fluorescence indicated interaction between the two fusion proteins. Positive and negative controls testing dimerization of PIP2-1 protein (Figure 4A) or interaction of NYFP/CKOR1 ([17]; Figure 4B) and NYFP/CYFP (Figure 4C) both yielded the expected results. The combination CESA7 and KOR1 only showed a background fluorescent signal (Figure 4E), whereas both CESA4 (Figure 4D) and CESA8 (Figure 4F) show a distinct signal when expressed in combination with KOR1. The interactions found *in planta* by the BiFC system confirmed the results found with the yeast two hybrid system.

In conclusion, KOR1 can interact with all the CESA proteins in the primary cellulose synthase complex *in planta*, but with only two of the secondary cellulose synthase complex proteins.

### The transmembrane domain is essential for interactions between KOR1 and CESA

The KOR1 protein is a membrane-anchored protein containing a short N-terminus located in the cytosol, a transmembrane domain (TMD), and an extracellular catalytic domain (Figure 1). In order to further characterize the interaction between KOR1 and the CESA proteins, several truncated KOR1 proteins were engineered. The N-terminal part of the protein together with the TMD was used to test whether the non-transmembrane portion of the protein (KOR1N; Figure 1B) is responsible for the interaction. The TMD with the C-terminal portion of the protein (KOR1C; Figure 1C) was tested for interactions between the catalytic domain and the CESA proteins. These truncated proteins were fused to the N-terminal portion of ubiquitin and used as prey, while the different CESA proteins were used as baits. All the truncated proteins of KOR1 containing the TMD, interacted with all CESA proteins, except for the CESA7 *in vitro* (Figure 2). Vice versa experiments with truncated KOR1 proteins used as bait and the different CESA proteins used as preys showed interaction except for CESA7 (Figure S1).

**Figure 5:**
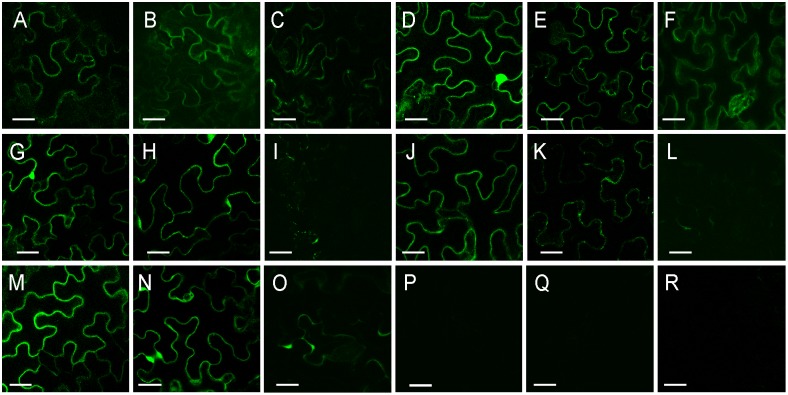
Bimolecular Fluorescence Complementation (BiFC) experiments in tobacco leaf epidermis. Confocal images are presented, showing YFP fluorescence indicating interaction. Tests for interactions between the CESAs and truncated versions of KOR1 are shown. (A) CESA1/KOR1N, (B) CESA1/KOR1C, (C) CESA1/KOR1TMD, (D) CESA3/KOR1N, E) CESA3/KOR1C, (F) CESA3/KOR1TMD, (G) CESA6/KOR1N, (H) CESA6/KOR1C, I) CESA6/KOR1TMD, (J) CESA4/KOR1N, (K) CESA4/KOR1C, (L) CESA4/KOR1TMD, (M) CESA8/KOR1N, (N) CESA8/KOR1C, (O) CESA8/KOR1TMD, (P) CESA7/KOR1N, (Q) CESA7/KOR1C, (R) CESA7/KOR1TMD. The results are representative of three independent experiments, n = 3. Scale bars = 100 µm.

To confirm these results *in planta*, truncated forms of KOR1 were tested for interaction with the primary (CESA1, CESA3, CESA6) and secondary (CESA4, CESA7, CESA8) CESAs using the BiFC assay. All the primary CESAs, CESA1, 3 and 6, were able to interact with KOR1N and KOR1C truncated proteins (Figure 5A–I). The fluorescent signal was comparable with that obtained for the full length KOR1 protein (Figure 4). Both CESA4 (Figure 5J–L) and CESA8 (Figure 5M–O) of the secondary CESAs were able to interact with the mentioned truncated forms of KOR1 (KOR1C and KOR1N) except for CESA7 which did not show interaction with any of the KOR1 constructs (Figure 5P–R). The fluorescent signal was comparable with that obtained for the full length KOR1 protein (Figure 4). The alternate pairwise combinations all resulted in similar interaction patterns except for KOR1C-CESA1.

As all the tested KOR1 domains containing the TMD were able to interact as well as the full length KOR1, it was deduced that the TMD might be involved in these interactions. To determine whether this domain is essential for the interaction, two recombinant proteins, one containing only the TMD and another, in which the TMD was absent were constructed to check for interaction (KOR1TMD AA 70–94, Figure 1D; KOR1NOTMD AA 1 to 69+95 to 621, Figure 1E). Because there are other glycosyl hydrolase family 9 members, which lack the TMD it was assumed that KOR1NOTMD was folded correctly. No interactions *in vitro* were found between the CESA proteins and KOR1NOTMD. Whereas KOR1TMD showed interactions with the primary CESAs (CESA1, CESA3 and CESA6) and also with CESA4 and CESA8 from the secondary cell wall (Figure 2). The reverse experiments with the KORTMD as bait and CESAs as prey also demonstrated the same interaction pattern (Fig. S1). Due to the split ubiquitin yeast two hybrid system used in these experiments being suitable for membrane bound proteins as bait, the interaction of KORNOTMD as bait with the CESAs as prey were not assessed. Although such experiments are technically possible, interpretation of negatives results is inconclusive.

To confirm these results *in planta*, KOR1TMD was tested for interaction with the primary (CESA1, CESA3, CESA6) and secondary (CESA4, CESA7, CESA8) CESAs using the BiFC assay. Confirming the in vitro assay results, the primary CESAs, CESA1, 3 were able to interact with the truncated protein KOR1TMD (Figure 5C, F, I) and CESA8 of the secondary CESAs were able to interact with the truncated KOR1TMD (Figure 5O), although signal intensity was significantly lower than that of the interactions with the full length KOR1. However, the combination of CESA6 from the primary and CESA4 from the secondary CESAs with KORTMD showed an extremely weak signal slightly stronger than a background fluorescent signal (Figure 5I and L). CESA7 did not show interaction with KOR1TMD (Figure 5R). The alternate pairwise combinations all resulted in similar interaction patterns (Figure S2). For the most part, the interactions found *in planta* by the BiFC system confirmed the results found with the yeast two hybrid system except for CESA6 and CESA4 which showed a very weak signal *in planta*.

### KORRIGAN1 is able to form homodimers *in planta*

**Figure 6:**
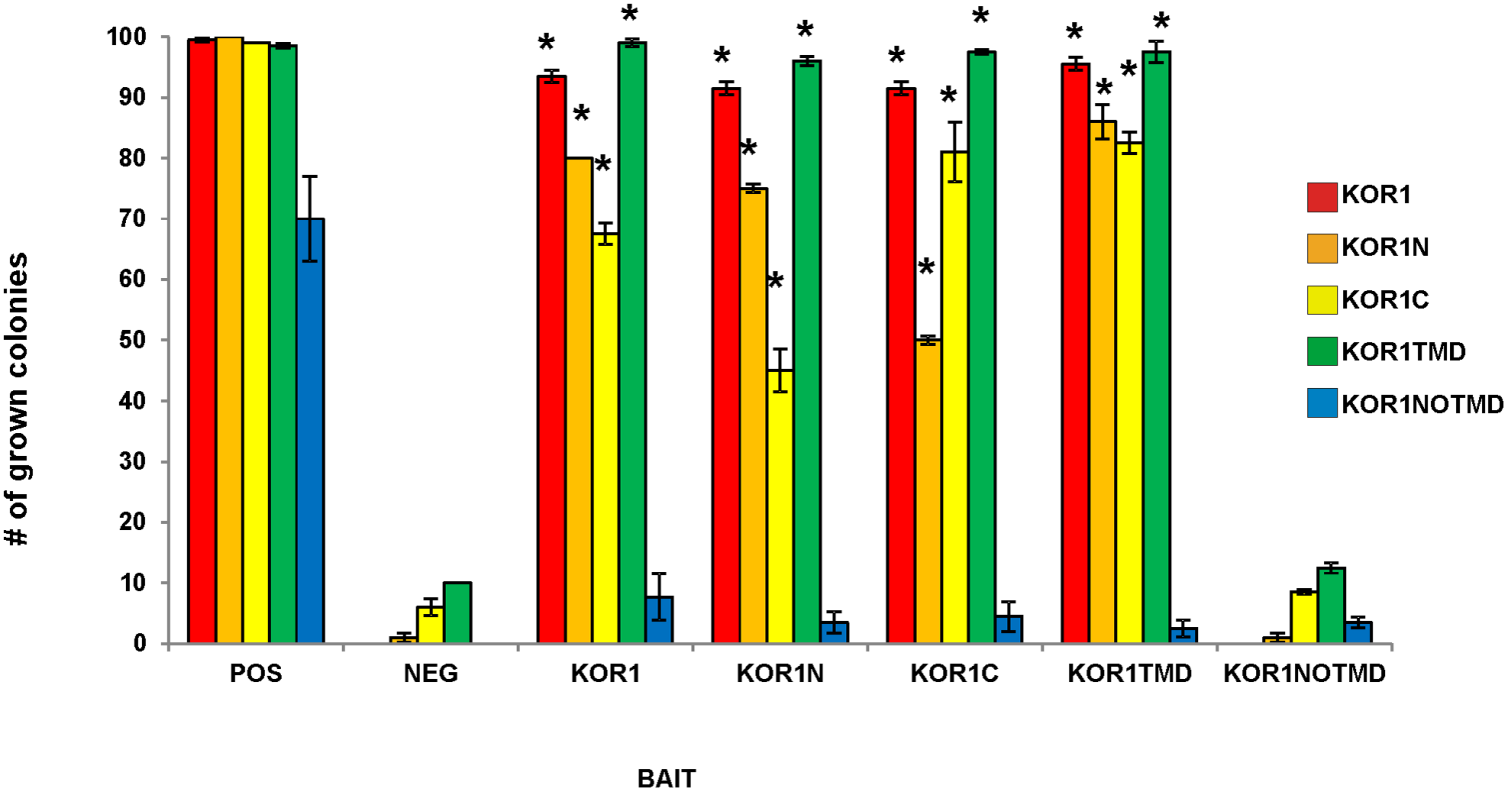
Interactions between the different KOR1 domains using the Membrane-based Yeast Two Hybrid. The bars represent the percentage of yeast colonies grown for 3 days on selective medium at 30°C. KOR1 domains represented in the legend were expressed in yeast as bait and the different KOR1 domains indicated at the x-axis as prey. ALG5 protein fused to wild-type ubiquitin domain was used as a positive control (POS) and NubG, fused to mutated ubiquitin domain as neg. control (NEG). The results are representative of three independent experiments, n = 3, Asterisks indicate statistically significant differences compared with the negative control (Student’s *t* test, *P<0.05).

It is known that type II membrane proteins, like KOR1, often form dimers to perform their function. Using the MbYTH assay, KOR1 was expressed both as bait and as prey to test the ability of KOR1 to form homodimers or higher order oligomers. The results showed that KOR1 could form homodimers, as it resulted in the growth of yeast on selective medium. To test whether a protein domain can be found responsible for this interaction the truncated KOR1 proteins were tested for interaction with each other. Growth was found in all combinations of truncated proteins except for those which lack a TMD. The combination of KOR1C as bait with KOR1N as prey, showed a weak yet significantly higher growth than its negative control background (KOR1C with the unrelated protein AGL5) (Figure 6).

**Figure 7:**
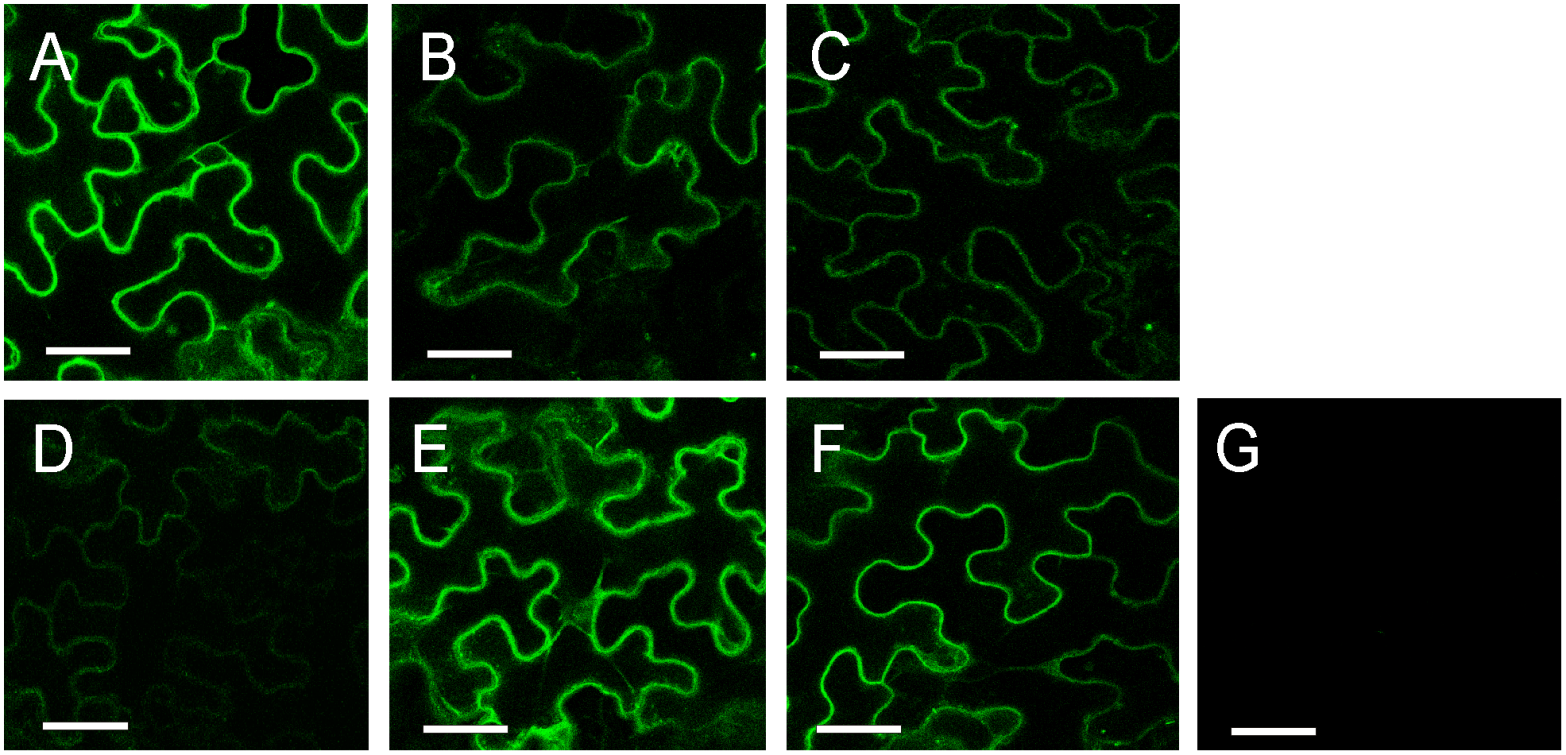
BiFC in *N. benthamiana* shows *in vitro* dimerization between KORRIGAN domains and the CESA1 protein. Dimerization between the different truncated forms of KORRIGAN: (A) KOR1/KOR1, (B) KOR1/KOR1N, (C) KOR1/KOR1C, (D) KOR1N/KOR1C, (E) KOR1N/KOR1N, (F) KOR1C/KOR1C, (G) negative control YN/YC. The results are representative of three independent experiments, n = 3. The scale bar is 100 µm.

To confirm these findings the interactions were tested *in planta.* Our results showed that fluorescence was restored when two different fusion proteins (YFP/C-KOR1 and YFP/N-KOR1) were expressed in *Nicotiana benthamiana*, indicating the formation of homodimers or higher oligomers of KOR1 proteins (Figure 7A). Different parts of the protein, KOR1N or KOR1C (Figure 1B and C), were also tested for interaction with the full-length or portions of KOR1 protein and fluorescence was found in all combinations tested. The partial proteins KOR1N and KOR1C could interact with the full-length KOR1 protein (Figure 6E and F, respectively) as well as with the respective truncated forms (Figure 6G) and form homodimers or higher oligomers (Figure 6H and I, respectively), although the interaction between the KOR1N and KOR1C (Figure 6G) was significantly weaker. As all (truncated) proteins in this test contained the TMD, these results indicate that this domain is also important in the dimerization of KOR1.

## Discussion

KORRIGAN1 has shown to play an important role in cellulose biosynthesis as the knockouts out of this gene results in reduced cellulose content. Despite previous studies indicating that some primary CESAs do not co-immunoprecipitate with KOR1 [24], more sensitive techniques such as split-ubiquitin assays and BiFC have been used to demonstrate direct interaction between KOR1 and all the primary cellulose synthases (CESA1, CESA3 and CESA6) [14], [16]. The same methodologies were used to assess whether interactions could be detected between KOR1 and the secondary CESAs. The methodology used here to assess the strength of interaction between KOR1 and the CESAs with the MbY2H is based on quantifying the number of fully grown colonies onto selective media. Despite only considering the fully grown colonies as interactions, the exact number of cells based on the colony size was not measured. It is important to note that assessing interaction strength with all protein-protein interaction methodologies available to date is somewhat difficult as the majority are not quantitative, but rather demonstrate the presence or lack of interaction. The results with our experimental procedures both *in vitro* and *in planta* demonstrated that KOR1 can interact with the three primary (CESA1, 3 and 6) only two of the secondary CESAs (CESA4 and 8), suggesting possible specificity for the different CESAs from the primary and secondary cell wall.

The alterations in cellulose content in the *kor1-1* dwarf mutant [1] together with the result that KOR1 directly interacted with CESA1, 3 and 6 indicated that KOR1 is an additional component of the CSC and is directly involved in the synthesis of cellulose in the primary cell wall [14]. The dwarf mutant *korrigan*, (*kor1-1*) phenotype results in a severe reduction in crystalline cellulose both in the primary and secondary cell wall [1] underlining the importance of glucan trimming during cellulose biosynthesis. The movement of cellulose synthase complex was reported to be slower in *kor1-3* and *kor1-1* mutants than in controls [14], [29]. It was also demonstrated that the *acw1* mutation effects cellulose accumulation in the cell wall and microfibril formation resulting in 40% reduction in crystalline cellulose content compared to wild type plants.

It must also be mentioned that the *kor1-1* mutant that showed distinct primary cell wall defects also showed signs of severely collapsed xylem similar to the *irx2* mutants in the secondary cell wall [16], showing that KOR1 is also involved in the secondary cell wall. The interaction with CESA4 and CESA8 indicates that KOR1 is part of the rosette structure of the secondary cell wall. The fact that CESA7 didn’t interact with KOR1 is supported by previous co-immunoprecipitation experiments, using an epitope-tagged form of KOR or IRX3 (AtCesA7) where KOR did not co-purify with AtCesA7 [16]. By checking the interactions of the other CESAs in the secondary cell wall (CESA4 and CESA8) with KOR1 both *in vitro* and *in planta* we demonstrate that there is indeed a physical interaction between KOR1 and the CESAs in the secondary cell wall except for CESA7. Other reports have also indicated that KOR plays a role in secondary cell wall development. Co-expression of *Populus* KOR orthologue with the three secondary cell wall CESA proteins has also been reported [30], [32]. It was also shown that over-expression of the putative *Atkor1* orthologue in hybrid aspen (PttCel9A1) causes a decrease in cellulose crystallinity [6]. Furthermore, two *irx2-1* and *irx2-2* KOR1 mutants showed reduced cellulose synthesis (30% of the WT) in the secondary cell wall [16] similar to the *irx* mutants. PtKOR1 is also shown to be required for secondary cell wall cellulose formation. RNAi suppression of PtKOR1 revealed a significant decrease in crystalline cellulose content and a reduced secondary cell wall thickness [33].

Interestingly, not all the CESAs in the primary and secondary cell wall have a similar interaction pattern with KOR1. The different reaction between the primary and the secondary cell wall proteins is difficult to explain as the specific functions of the different CESA proteins are not known. As previously mentioned CESA1 was the most efficient interactor in the primary cell wall whereas CESA7 from the secondary cell wall did not interact with KOR1 suggesting that specific CESA isoforms may have unique roles in recruiting KOR1. In other words the binding of KOR1 to the different CESA proteins is specific, as KORRIGAN1 does not bind to all of them. Not only does this imply that the methods used are sensitive enough to specifically determine interactions between these highly homologous proteins, it also indicates that the KOR1 protein has a specific position within the rosette.

A more detailed view on the interaction between KOR1 and the CESA proteins using truncated versions of KOR1 revealed that all portions which contain the TMD were able to interact and this led to the conclusion that KOR1 transmembrane domain is required for the interaction with CESAs. These data suggest that KOR1 and CESA associate in the CSC in the plasma membrane, between the TMD of KORRIGAN and the TMDs of the CESA proteins. Surprisingly, despite showing interaction *in vitro*, CESA6 and CESA4 show a very weak to no interaction with KOR1TMD *in planta* suggesting that at least for the two mentioned CESAs the presence of TMD is essential but not sufficient for interaction and would require more than just the TMD. It is interesting to note that some proteins required for cellulose synthesis are found in sterol-rich lipid rafts [34]. It is possible that KOR1 and CESAs traffic to the plasma membrane separately, but are then concentrated in specific lipid domains, favoring interactions. This domain is also important in the homodimerisation of the KOR1 protein as all partial proteins containing this domain are able to interact, however other domains of the KOR1 protein might also play a role in the dimerization as the combination KOR1C and KOR1N only showed a weak interaction in both assays. The function of this dimerization is thus far unknown, however one could speculate that the dimer enables a more efficient hydrolysis of the glucan chains or binding to the cellulose, or the interlinking of KORRIGAN might result in a more stable rosette complex.

In conclusion, we have determined that the KOR1 protein interacts with secondary cellulose synthase complex proteins both *in vitro* and *in planta* supporting the hypothesis that KOR1 makes up a part of the rosette structure. The physical interaction also indicates that KOR1 is directly involved in cellulose biosynthesis, and probably does so in the form of a homodimer or a higher order oligomer. Furthermore, our study showed that the TMD of KOR1 is essential not only in the interaction with the different CESA proteins, but also for KOR1 homodimerization.

## Supporting Information

Figure S1 **Interactions between the different KOR1 domains and the different CESA proteins using the Membrane-based Yeast Two Hybrid.** The bars represent the percentage of yeast colonies grown for 3 days on selective medium at 30°C. The different CESA proteins were expressed in yeast as prey and the different KOR1 protein domains as bait.
(PDF)

Figure S2 **Bimolecular Fluorescence Complementation (BiFC) experiments in tobacco leaf epidermis.** Confocal images are presented, showing YFP fluorescence indicating interaction. Tests for interactions between the CESAs and truncated versions of KOR1 are shown (A) KOR1N/CESA1, (B) KOR1C/CESA1, (C) KOR1TMD/CESA1, (D) KOR1N/CESA3, (E) KOR1C/CESA3, (F) KOR1TMD/CESA3, (G) KOR1N/CESA6, (H) KOR1C/CESA6, (I) KOR1TMD/CESA6, (J) KOR1N/CESA4, (K) KOR1C/CESA4, (L) KOR1TMD/CESA4, (M) KOR1N/CESA8, (N) KOR1C/CESA8, (O) KOR1TMD/CESA8, (P) KOR1C/CESA7, (Q) KOR1N/CESA7, (R) KOR1TMD/CESA7. Scale bars = 100 µm.
(PDF)

Table S1 **Primers used for the MbYTH system.**
(PDF)
