# Correction: KORRIGAN1 Interacts Specifically with Integral Components of the Cellulose Synthase Machinery

**DOI:** 10.1371/journal.pone.0140411

**Published:** 2015-10-07

**Authors:** Nasim Mansoori, Jaap Timmers, Thierry Desprez, Claire L. Alvim-Kamei, Dianka C. T. Dees, Jean-Paul Vincken, Richard G. F. Visser, Herman Höfte, Samantha Vernhettes, Luisa M. Trindade

The fourth author’s name is spelled incorrectly. The correct name is: Claire L. Alvim-Kamei. The correct citation is: Mansoori N, Timmers J, Desprez T, Alvim-Kamei CL, Dees DCT, Vincken J-P, et al. (2014) KORRIGAN1 Interacts Specifically with Integral Components of the Cellulose Synthase Machinery. PLoS ONE 9(11): e112387. doi:10.1371/journal.pone.0112387
