# Supplementary material for: KORRIGAN1 Interacts Specifically with Integral Components of the Cellulose Synthase Machinery
Source: PLoS One. 2014 Nov 10;9(11):e112387. doi: 10.1371/journal.pone.0112387 (PMC4226561; doi:10.1371/journal.pone.0112387)
Supplement: Figure S1 — Interactions between the different KOR1 domains and the different CESA proteins using the Membrane-based Yeast Two Hybrid. The bars represent the percentage of yeast colonies grown for 3 days on selective medium at 30°C. The different CESA proteins were expressed in yeast as prey and the different KOR1 protein domains as bait. (PDF) [file pone.0112387.s001.pdf]

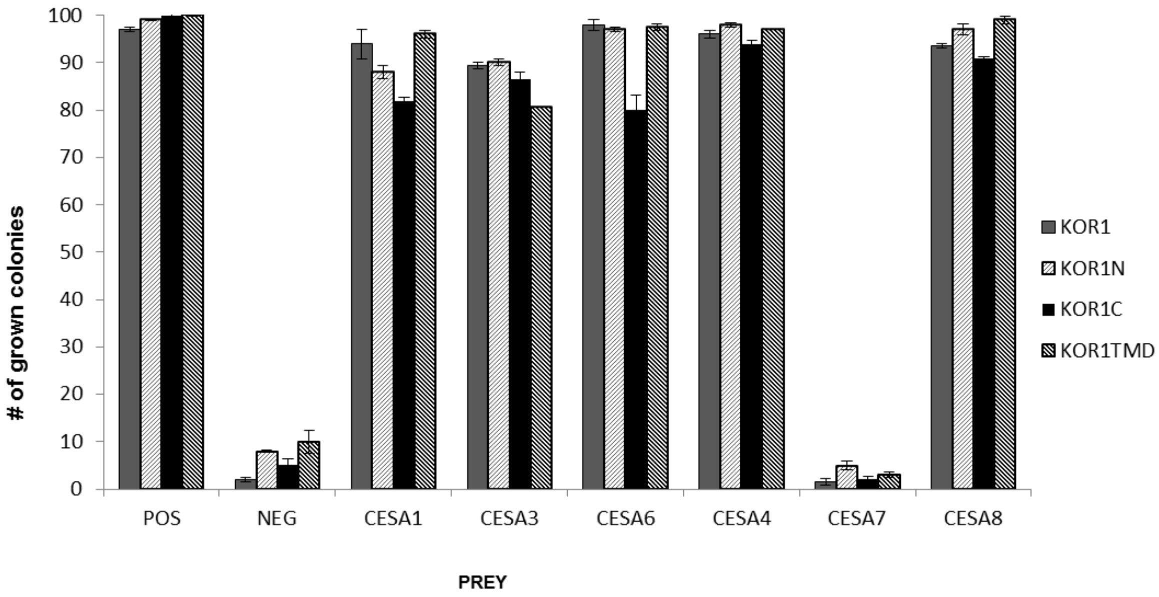

Figure S1. Interactions between the different KOR1 domains and the different CESA proteins using the Membrane-based Yeast Two Hybrid. The bars represent the percentage of yeast colonies grown for 3 days on selective medium at 30 ºC. The different CESA proteins were expressed in yeast as prey and the different KOR1 protein domains as bait.
