# Supplementary material for: KORRIGAN1 Interacts Specifically with Integral Components of the Cellulose Synthase Machinery
Source: PLoS One. 2014 Nov 10;9(11):e112387. doi: 10.1371/journal.pone.0112387 (PMC4226561; doi:10.1371/journal.pone.0112387)
Supplement: Figure S2 — Bimolecular Fluorescence Complementation (BiFC) experiments in tobacco leaf epidermis. Confocal images are presented, showing YFP fluorescence indicating interaction. Tests for interactions between the CESAs and truncated versions of KOR1 are shown (A) KOR1N/CESA1, (B) KOR1C/CESA1, (C) KOR1TMD/CESA1, (D) KOR1N/CESA3, (E) KOR1C/CESA3, (F) KOR1TMD/CESA3, (G) KOR1N/CESA6, (H) KOR1C/CESA6, (I) KOR1TMD/CESA6, (J) KOR1N/CESA4, (K) KOR1C/CESA4, (L) KOR1TMD/CESA4, (M) KOR1N/CESA8, (N) KOR1C/CESA8, (O) KOR1TMD/CESA8, (P) KOR1C/CESA7, (Q) KOR1N/CESA7, (R) KOR1TMD/CESA7. Scale bars = 100 µm. (PDF) [file pone.0112387.s002.pdf]

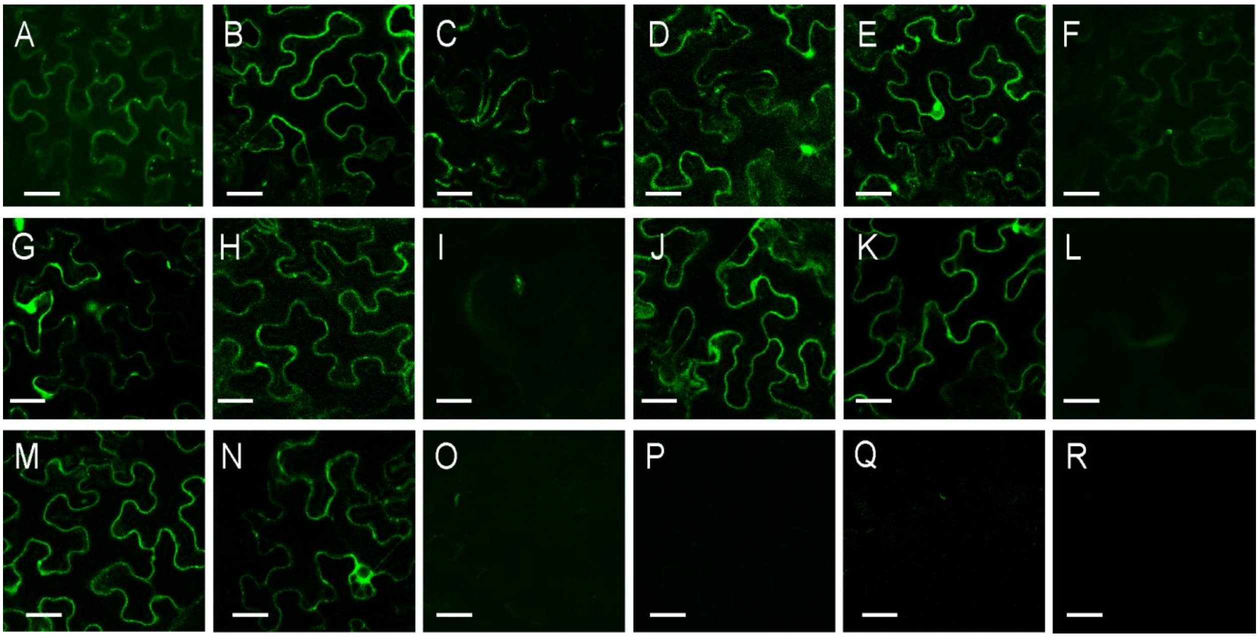

Figure S2: Bimolecular Fluorescence Complementation (BiFC) experiments in tobacco leaf epidermis. Confocal images are presented, showing YFP fluorescence indicating interaction. Tests for interactions between the CESAs and truncated versions of KOR1 are shown (A) KOR1N/CESA1,(B) KOR1C/CESA1, (C) KOR1TMD/CESA1, (D) KOR1N/CESA3, (E) KOR1C/CESA3, (F) KOR1TMD/CESA3, (G) KOR1N/CESA6, (H) KOR1C/CESA6, (I) KOR1TMD/CESA6, (J) KOR1N/CESA4, (K) KOR1C/CESA4, (L) KOR1TMD/CESA4, (M) KOR1N/CESA8, (N) KOR1C/ CESA8, (O) KOR1TMD/CESA8, (P) KOR1C/CESA7, (Q) KOR1N/CESA7, (R) KOR1TMD/CESA7,. Scale bars = 100 µm.
