# Supplementary material for: KORRIGAN1 Interacts Specifically with Integral Components of the Cellulose Synthase Machinery
Source: PLoS One. 2014 Nov 10;9(11):e112387. doi: 10.1371/journal.pone.0112387 (PMC4226561; doi:10.1371/journal.pone.0112387)
Supplement: Table S1 — Primers used for the MbYTH system. (PDF) [file pone.0112387.s003.pdf]

## Table S1: Primers used for the MbYTH system

| **Gene** | **Primer for bait amplification** |
|---|---|
| CESA1 | 5’AAGACTGCAGAATGGAGGCCAGTGCCGGC3’<br>5’AACAGGCGCCCTAAAAGACACCTCCTTTGCC3’ |
| CESA3 | 5’AGAACCATGGAATGGAATCCGAGGAGAAACC3’<br>5’AAGAACTAGTTCAACAGTTGATTCCACTTCC3’ |
| CESA6 | 5’AGAACCATGGAATGAACACCGGTGGTCGG3’<br>5’AAGAACTAGTTCACAAGCAGTCTAAACCA3’ |
| KOR1 | 5’AAGACGTCATGTACGGAAGAGATCCATGGGG3’<br>5’TTTACTAGTCAAGGTTTCCATGGTGCTGGTGG3’ |
| KOR1C | 5’AAAGACGTCAAGATCTTCGTCTGGACTGTTGGT3’<br>5’TTTACTAGTCAAGGTTTCCATGGTGCTGGTGG3’ |
| KOR1N | 5’AAGACGTCATGTACGGAAGAGATCCATGGGG3’<br>5’TTTACTAGTTTAACGATCAAGGTAATGAA3’ |
| KOR1 TMD | 5’AAAGACGTCAAGATCTTCGTCTGGACTGTTGGT3’<br>5’TTTACTAGTTTAACGATCAAGGTAATGAA3’ |
| KOR1 NOTMD | 5’AAGACGTCATGTACGGAAGAGATCCATGGGG3’<br>5’GCGGCTAACGATAATACAACC3’/5’ACTGTGCCGCGTCATCATCC3’<br>5’ACTGTGCCGCGTCATCATCC3’<br>5’TTTACTAGTCAAGGTTTCCATGGTGCTGGTGG3’ |
| **Gene** | **Primer for prey amplification** |
| CESA1 | 5’AAGAGGCCATTACGGCCATGGAGGCCAGTGCCGGC3’<br>5’AAGAGGCCGAGGCGGCCAAGTAAAAGACACCTCCTTTGCCAT3’ |
| CESA3 | 5’AGAACGGCCATTACGGCCATGGAATCCGAAGGAGAAACC3’<br>5’GAGGCCGAGGCGGCCGTCAACAGTTGATTCCACATTCCAGAAT3’ |
| CESA6 | 5’AGAACGGCCATTACGGCCATGAACACCGGTGGTCGGTTAATCGC3’<br>5’GAGGCCGAGGCGGCCGTCACAAGCAGTCTAAACCACAGATCTCGAGAAT3’ |
| KOR1 | 5’AACAGGCCATTACGGCCATGTACGGAAGAGATCCATGGGG3’<br>5’AAGAGGCCGAGGCGGCCATCAAGGTTTCCATGGTGCTGGTGG3’ |
| KOR1C | 5’AACAGGCCATTACGGCCAAGATCTTCGTCTGGACTGTTGGT3’<br>5’AAGAGGCCGAGGCGGCCATCAAGGTTTCCATGGTGCTGGTGG3’ |
| KOR1N | 5’AACAGGCCATTACGGCCATGTACGGAAGAGATCCATGGGG3’<br>5’AAGAGGCCGAGGCGGCCTTTAACGATCAAGGTAATGAA3’ |
| KOR1 TMD | 5’AACAGGCCATTACGGCCAA ’<br>5’AAGAGGCCGAGGCGGCC |
| KOR1 NOTMD | 5’AACAGGCCATTACGGCCATGTACGGAAGAGATCCATGGGG3’<br>5’GCGGCTAACGATAATACAACC3’<br>5’ACTGTGCCGCGTCATCATCC3’<br>5’AAGAGGCCGAGGCGGCCATCAAGGTTTCCATGGTGCTGGTGG3’ |
